# Chronic kidney disease increases the risks of age-related macular degeneration: a systematic review and meta-analysis

**DOI:** 10.3389/fmed.2025.1635766

**Published:** 2025-11-27

**Authors:** Ting-Han Lin, You-Yen Lin, Yu-Te Huang, Yu-Chuen Huang, Peng-Tai Tien, Lei Wan, Hui-Ju Lin

**Affiliations:** 1 Department of General Medicine, China Medical University Hospital, Taichung, Taiwan; 2 Division of Genetics and Metabolism, Children's Hospital of China Medical University, Taichung, Taiwan; 3 Department of Ophthalmology, Eye Center, China Medical University Hospital, Taichung, Taiwan; 4 School of Chinese Medicine, China Medical University, Taichung, Taiwan; 5 School of Medicine, China Medical University, Taichung, Taiwan; 6 Department of Medical Laboratory Science and Biotechnology, Asia University, Taichung, Taiwan; 7 Department of Obstetrics and Gynecology, China Medical University Hospital, Taichung, Taiwan

**Keywords:** chronic kidney disease, age-related macular degeneration, macular degeneration, age-related maculopathy, oxidative stress

## Abstract

**Importance:**

Age-related macular degeneration (AMD) and chronic kidney disease (CKD) are common causes of morbidity, with systemic risk factors shared. Clarifying the association between the two is crucial to guiding comprehensive management.

**Objective:**

This study aimed to systematically review current and latest evidence on the influence of CKD on AMD prevalence. An investigation was performed into various AMD stages and how different CKD severities exert their effects.

**Data sources:**

Databases of PubMed and Embase were searched from their inception to 11 November 2024. Reference lists of studies were reviewed, and relevant researchers were contacted. The study was accepted and registered with PROSPERO (CRD420250612669).

**Study selection:**

Eligible studies of the current review are observational, peer-reviewed, and include quantitative comparisons of AMD prevalence between populations with and without CKD. Studies with overlapping data or investigating AMD incidence were excluded. Twenty studies met the inclusion criteria from the 3,218 initially identified.

**Data extraction and synthesis:**

We extracted data and assessed study quality using the Appraisal tool for Cross-Sectional Studies (AXIS) tool for cross-sectional studies. Our meta-analyses followed the Preferred Reporting Items for Systematic Reviews and Meta-Analyses guidelines and were conducted with Review Manager 5.4.1. Risk of bias was evaluated using the AXIS quality appraisal tool, and the overall certainty of evidence was qualitatively assessed following the GRADE approach.

**Main outcomes and measures:**

Primary outcomes are the prevalence of all-stage, early-stage, and late-stage AMD among CKD and non-CKD patients. Secondary outcomes investigated AMD prevalence among patients with different CKD stages. We reported in our analyses the risk ratios (RRs) with 95% confidence intervals (CIs).

**Results:**

All-stage AMD prevalence was found to be higher among CKD patients (RR: 1.65; 95% CI: 1.49–1.83). Similarly, early-stage AMD was more prevalent among CKD patients (RR: 1.47; 95% CI: 1.26–1.71). In late-stage AMD, an even stronger association was shown (RR: 3.72; 95% CI: 2.14–6.45). Meanwhile, there was no significant difference in AMD prevalence between moderate and advanced CKD stages (RR: 1.08; 95% CI: 0.48–2.46).

**Conclusion and relevance:**

Our findings indicate that CKD is significantly associated with higher AMD prevalence. These findings suggest the effects of shared systemic mechanisms and underscore the need for ophthalmic screening in CKD patients. Further studies are needed to strengthen causality and expand generalizability beyond the Asian population.

**Systematic review registration:**

The systematic review was registered in PROSPERO (CRD420250612669).

## 1 Introduction

The macula is the part of the retina with the highest concentration of cone photoreceptor cells, which are fundamental to central vision (1). Age-related macular degeneration (AMD), or simply macular degeneration, affects one in eight individuals aged 60 years or older (2) and is recognized as one of the leading causes of irreversible blindness globally (3). Early AMD is manifested by a slow and progressive degeneration of the retinal pigment epithelium and photoreceptors, with drusen and pigmentary changes. Late AMD, on the other hand, is manifested by geographical atrophy (dry AMD) or neovascularization (wet AMD) (4, 5).

By the year 2021, more than 850 million people were estimated suffer from some form of kidney disease, as indicated by a joint statement from the American Society of Nephrology, European Renal Association, and International Society of Nephrology (6). According to the Kidney Disease: Improving Global Outcomes (KDIGO) 2024 Clinical Practice Guideline, chronic kidney disease (CKD) is defined as abnormalities of kidney structure or function, present for a minimum of 3 months, with implications for health (6). Both as end-organ targets of systemic diseases, the comorbidity of the kidney and eye has been linked to factors such as diabetes, hypertension, and uremia (7, 8). As a result, the relationship between AMD and CKD has become a focus of research in many studies. CKD features the accumulation of uremic toxins (e.g., indoxyl sulfate and p-cresyl sulfate) that drive endothelial dysfunction, vascular calcification, and pro-inflammatory signaling in the vasculature (9, 10). In AMD, oxidative stress-driven retinal pigment epithelium (RPE) dysfunction with impaired phagocytosis/autophagy and disturbed angiogenic responses are key pathological processes (11). Mendelian randomization evidence further links systemic inflammatory regulators with AMD risk (12). Together, these systemic and retinal alterations provide a plausible mechanistic bridge between impaired renal clearance and retinal degeneration (13). Two previous reviews (3, 14) have provided insight into the correlation between the two diseases. However, there exists a need for a meta-analytic update since both reviews included studies that the other did not, and there are relevant studies never included in previous reviews (15, 16).

Hence, the objective of this systematic review and meta-analysis is to review the latest clinical evidence on the relationship between AMD and CKD (measured in eGFR) and to provide a comprehensive conclusion for relevant clinical care providers.

## 2 Methods

### 2.1 Data sources

Relevant studies were retrieved from PubMed and Embase databases that were published from the inception of the databases to 11 November 2024, irrespective of language. In addition, the Cochrane Library was screened to identify relevant systematic reviews and to cross-check reference lists for completeness. This supplementary search was reflected in the PRISMA flow diagram for transparency. However, systematic reviews themselves were not included in the quantitative synthesis; only original observational studies were analyzed.

While searching for eligible studies, we applied the following search terms and medical subject headings in various combinations: “macular degeneration,” “age-related macular degeneration,” “age-related maculopathy,” “kidney,” “renal,” and “chronic kidney disease.” Furthermore, the reference sections of retrieved studies were manually investigated, and known researchers with relevant expertise were contacted. The current systematic review and meta-analysis was prospectively registered in the PROSPERO International Prospective Register of Systematic Reviews (CRD420250612669). No separate review protocol was publicly posted prior to data collection, and no major deviations from the registered protocol were made during the review process. PICOS question and objective. The research question was structured according to the PICOS framework: P (Population): adults with chronic kidney disease (CKD); I (Exposure): reduced renal function measured by estimated glomerular filtration rate (eGFR) or a clinical diagnosis of CKD; C (Comparator): individuals without CKD or with normal renal function; O (Outcome): presence or stage of age-related macular degeneration (AMD); and S (Study design): observational studies (cross-sectional, case–control, or cohort). Accordingly, the objective of this review was to quantify the association between CKD and AMD across disease stages using meta-analytic synthesis. Definition of eGFR. The term eGFR refers to estimated glomerular filtration rate, a standard indicator of kidney function derived from serum creatinine-based equations [e.g., Chronic Kidney Disease Epidemiology Collaboration (CKD-EPI) or Modification of Diet in Renal Disease (MDRD)].

With regard to studies from the Asian Eye Epidemiology Consortium (AEEC), data were derived from a previously published systematic review by Rim et al., which synthesized epidemiologic findings from the AEEC cohorts. From this review, 10 eligible studies were identified and included in our meta-analysis (17).

### 2.2 Study selection

We included studies that provided a quantitative comparison of AMD prevalence between populations with and without CKD. We excluded studies based on the following criteria: overlapping patient cohorts in two or more studies with identical outcomes or studies focused on the incidence of AMD.

All records were screened independently at each stage following predefined eligibility criteria. Disagreements during study selection were resolved through consensus, with arbitration applied when necessary. Data extraction was conducted independently and cross-checked to ensure consistency and accuracy.

### 2.3 Data extraction and quality assessment

Potentially eligible studies were initially screened based on their article titles and abstracts. Then, their full-text versions were retrieved for further evaluation. Two independent reviewers (Ting-Han Lin and Peng-Tai Tien) assessed studies for inclusion. They extracted the study data using a standardized procedure, while a third reviewer (Lei Wan) assisted in resolving disagreements and discrepancies. We contacted the study authors for additional information as needed.

Data on the study design, patient characteristics (e.g., gender, age, and diagnosis of AMD), the definition of CKD, and analytical outcomes were extracted. Using the Appraisal tool for Cross-Sectional Studies (AXIS), Ting-Han Lin performed the risk-of-bias analysis for the included studies. Extracted variables also included sample size, geographic region, study year, AMD classification (early, late, or overall), CKD stage based on eGFR thresholds, and laboratory indicators such as serum creatinine or estimated glomerular filtration rate (eGFR) used to define CKD status.

### 2.4 Data synthesis and analysis

Studies that investigated the relationship between the disease status of CKD and AMD were assessed in separate meta-analyses. Since there are studies that did not explicitly indicate their definition of CKD, we universally applied “eGFR <60” to the CKD group and “eGFR ≥60” to the non-CKD group (shown in Table 1). Subgroups were introduced to provide elucidation on the effects of CKD on the various stages of AMD and whether the severity of CKD affects the prevalence of AMD. Our primary outcomes include the comparisons between CKD and non-CKD patients on the prevalence of all-stage, early-stage, and late-(advanced)stage AMD. Our secondary outcome compared between the moderate stage and advanced stage of CKD on the prevalence of AMD.

**Table 1:** Characteristics of included trials.

**Author [Year]**	**Study design**	**Male, n (%)**	**Age, year, mean ± SD**	**Diagnosis of AMD**	**Type of AMD**	**Exposure (CKD Definition)**	**Control**
Neelamegam 2024	Cross-sectional	eGFR >60: 1944 (45.9) eGFR <60: 166 (29.8)	eGFR >60: 65.49 ± 5.98 eGFR <60: 68.40 ± 7.01	Grading as per the International ARM Epidemiological Study Group	Early, Dry, Wet	eGFR <60 (Moderate to severe CKD)	eGFR >60 (No to mild CKD)
Jung 2023	Data used: Cross-sectional; Initial design: Retrospective cohort	Non-AMD: 2013798 (48.5) AMD: 22783 (42.5)	Non-AMD: 60.6 ± 8.3 AMD: 67.4 ± 8.4	ICD-10	AMD with VD, AMD without VD	†eGFR <60	†eGFR ≥60
Zhu 2020	Cross-sectional	2762 (50.1)	56.9 ± 0.40 (SE)	Modified Wisconsin Age-Related Maculopathy Grading Classification Scheme	Any	eGFR <60	eGFR ≥60
Chen 2017	Cross-sectional	79,175 (51.59)	54.9 ± 15.7	ICD-9	Any, advanced	Adult w/ ICD-9 diagnosis of CKD (excluding advanced CKD, dialysis or renal transplantation)	Non-CKD
Chong 2014	Cross-sectional	Cystatin C level Top decile: 367 (51.6) Bottom nine deciles: 2858 (46.8)	Cystatin C level Top decile: 70.0 ± 9.5 Bottom nine deciles: 61.4 ± 10.0	**Early:** either presence of any soft drusen and pigmentary abnormalities or reticular drusen, in the absence of signs of late end-stage AMD	Early	eGFR ≤60	eGFR >60
Choi 2011	Cross-sectional	Non-CKD: 1482 (62.4) CKD: 343 (54.2)	Non-CKD: 56.7 ± 6.3 CKD: 62.1 ± 7.5	Modification of the Wisconsin AMD Grading System	Early	eGFR ≤60	eGFR >60
Deva 2011	Cross-sectional	CKD Stages 3 to 5: 96 (64) CKD Stages 1 to 2: 96 (64)	Median=62 (range 20 to 85)	Graded by an ophthalmologist and trained observer	Any, severe	CKD Stages 3 to 5 (eGFR <60 for at least 3 mth)	CKD Stages 1 to 2 (eGFR ≥60)
Gao 2011	Cross-sectional	5941 (61.6)	52.8 ± 16.0	Large drusen and pigmentary changes on ophthalmoscopes	Any	eGFR <60 and/or proteinuria	Non-CKD
Weiner 2011	Cross-sectional	3695 (48.2)	59.3 ± 13.1	**Early:** either soft drusen (≥63μm, consistent w/ Grade 3 drusen in WARMGS) or any drusen type w/ areas of depigmentation/hypopigmentation of retinal pigment epithelium w/o any visibility of choroidal vessels or w/ increased retinal pigment in macular area **Late:** signs of exudative macular degeneration or geographic atrophy	Early, Late	†eGFR <60	†eGFR ≥60
Nitsch 2009	Cross-sectional	1292 (44.9)	N/A (aged 75 years or over)	Did not mention		eGFR <60	eGFR ≥60
BES	Cross-sectional	646 (41.3)	64.4**±** 9.6	Modified WARMGS	Any, early, late	eGFR <60	eGFR ≥60
CIEMS	Cross-sectional	2160 (46.5)	49.4**±** 13.4	Modified WARMGS	Any, early, late	eGFR <60	eGFR ≥60
HDES	Cross-sectional	2089 (44.8)	50.9**±** 10.6	Modified WARMGS	Any, early, late	eGFR <60	eGFR ≥60
KNHANES	Cross-sectional	7374 (43.0)	57.5**±** 11.4	International classification and grading system for age-related maculopathy and AMD	Any, early, late	eGFR <60	eGFR ≥60
SCES	Cross-sectional	1580 (50.1)	59.3**±** 9.7	Modified WARMGS	Any, early, late	eGFR <60	eGFR ≥60
SiMES	Cross-sectional	1464 (48.3)	58.8**±** 10.9	Modified WARMGS	Any, early, late	eGFR <60	eGFR ≥60
SINDI	Cross-sectional	1617 (50.9)	57.4**±** 9.9	Modified WARMGS	Any, early, late	eGFR <60	eGFR ≥60
SNDREAMS	Cross-sectional	2110 (44.0)	65.8**±** 6.2	Beckman Initiative for Macular Research	Any, early, late	eGFR <60	eGFR ≥60
TMCS	Cross-sectional	2154 (54.3)	62.4**±** 7.5	Modified WARMGS	Any, early, late	eGFR <60	eGFR ≥60
UEMS	Cross-sectional	2044 (40.1)	58.7**±** 10.6	Beckman Initiative for Macular Research	Any, early, late	eGFR <60	eGFR ≥60

Abbreviations: ARM: age-related maculopathy; BES = Beijing Eye Study; CIEMS = Central India Eye and Medical Study; eGFR: estimated glomerular filtration rate (unit in ml/min/1.73 m^2^) ; HDES = Handan Eye Study; ICD- 9=International Classification of Diseases, Ninth Revision; ICD-10=International Classification of Diseases, Tenth Revision; KNHANES = Korean National Health and Nutrition Examination Survey; SiMES = Singapore Malay Eye Study; SINDI = Singapore Indian Eye Study; SCES = Singapore Chinese Eye Study; SNDREAMS = Sankara Nethralaya-Diabetic Retinopathy Epidemiology and Molecular Genetic Study; TMCS = Tsuruoka Metabolomics Cohort Study; UEMS = Ural Eye and Medical Study; VD = visual disability; WARMGS: Wisconsin Age-related Maculopathy Grading System

† Defined by the current study.

We calculated risk ratios (RRs) by dividing the prevalence of AMD in the CKD (or advanced CKD) group by that in the non-CKD (or moderate CKD) group. We also computed 95% confidence intervals (CIs) for these values. We used the Review Manager statistical package (version 5.4.1; The Cochrane Collaboration, 2020) to conduct all analyses. We followed the guidelines of the Preferred Reporting Items for Systematic Reviews and Meta-Analyses (18). To assess heterogeneity across the studies, chi-square statistics and *I*^2^ tests were used. Random-effects models (DerSimonian–Laird) were applied when significant heterogeneity was detected (*I*^2^ > 50%); otherwise, fixed-effects models were used. Subgroup analyses (by ethnicity and study design) and leave-one-out sensitivity analyses were performed to assess robustness. Publication bias was examined using funnel plots and Egger’s test. The overall certainty of evidence was evaluated according to the GRADE framework, considering consistency, precision, and directness of results. Potential publication bias was assessed through the visual inspection of funnel plots and Egger’s regression test, although the limited number of included studies precluded definitive conclusions. The certainty of evidence for each outcome was qualitatively appraised based on consistency, precision, and directness of results, referencing the GRADE framework. As all included studies were observational, the overall certainty was rated as low to moderate.

## 3 Results

### 3.1 Characteristics of the included studies

**Figure 1:**
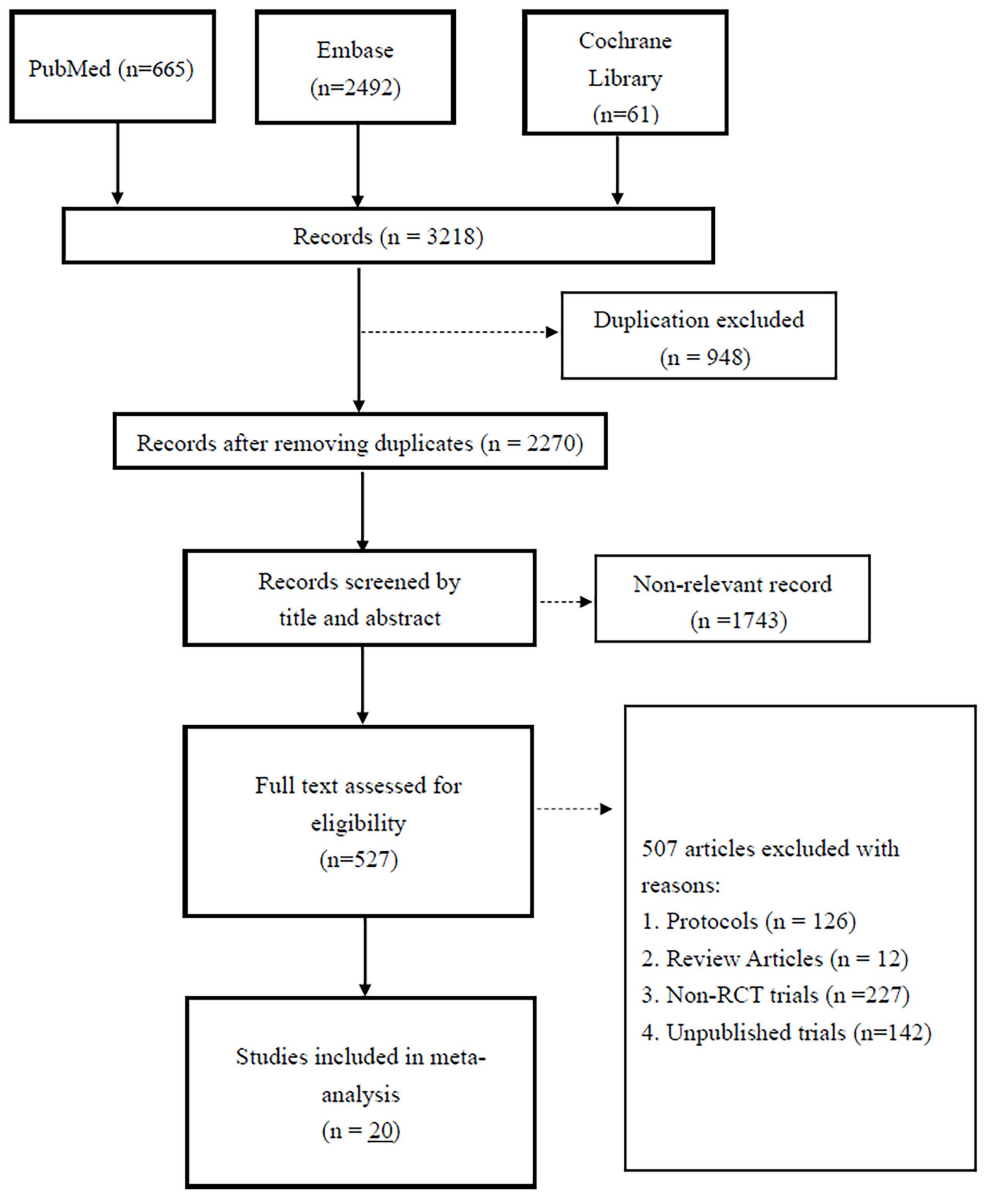
PRISMA flow diagram of study selection. Summary of identification, screening, eligibility assessment, and final inclusion of 20 observational studies evaluating the association between chronic kidney disease (CKD) and age-related macular degeneration (AMD).

Our initial search yielded 3,218 articles, of which 948 were duplicate records and were excluded (Figure 1). Screening excluded 1,743 articles that were judged to be non-relevant. Of the 527 remaining articles, 507 failed to meet the inclusion criteria and were excluded. Therefore, 20 studies were eligible for our meta-analysis (3, 15, 16, 19–26).

All except one (16) included studies are cross-sectional studies. While the one different study (16) was initially designed as a retrospective cohort; their data were applicable in our analysis as a cross-sectional study. Therefore, all included studies were analyzed as cross-sectional studies (Table 1). Most of the included studies stated their compliance with the Declaration of Helsinki, except for three studies (27–29). As Chen (29) used deidentified secondary data from the National Health Insurance (NHI) dataset; the study was exempted from institutional review board review.

Notably, 10 studies, including the Beijing Eye Study and Singapore Chinese Eye Study, were within the Asian Eye Epidemiology Consortium (AEEC). Since the majority of the data were provided to the AEEC but not published, we retrieved them from a previously conducted systematic review (3).

### 3.2 Risk-of-bias assessment

An evaluation of the methodological quality of the included studies using the AXIS tool’s 20-item checklist can be found in Table 2. Each study was assessed across criteria such as clarity of objectives, representativeness of the population base, adequacy of statistical reporting, and reporting of ethical approval and patient consent. While most studies demonstrate strengths in fields such as clearly defined objectives and appropriate study designs, their common limitation is most remarkable in the lack of justification for sample size. Notably, 10 studies within the AEEC were marked with “Not Applicable” across many items, reflecting the limited availability of the unpublished data.

**Table 2:** Risk-of-bias assessment with AXIS (color-coded summary of individual studies).

**Author [Year]**	Clear objectives?	Study design appropriate?	Sample size justified?	Target population clearly defined?	Population base representative?	Selection process representative?	Measures for non-responders	Appropriate risk factors and outcomes?	Risk factors and outcomes measured correctly?	Clear statistical significance and precision estimates?	Methods sufficiently described	Basic data adequately described	Non-response bias†	Information about non-responders	Results internally consistent	Results of analyses presented	Discussions and conclusions justified by results	Limitations discussed?	Conflicts of interest	Ethical approval and consent
Neelamegam 2024	Y	Y	N	Y	?	?	N	Y	?	Y	Y	Y	NA	NA	Y	Y	Y	Y	N	Y
Jung 2023	Y	Y	N	?	?	?	NA	Y	?	Y	NA	NA	NA	NA	Y	NA	Y	Y	N	Y, NA
Zhu 2020	Y	Y	N	Y	Y	Y	NA	Y	Y	Y	Y	N	NA	NA	Y	Y	Y	Y	N	Y
Chen 2017	Y	Y	N	Y	?	?	NA	Y	Y	Y	Y	N	N	NA	Y	Y	Y	Y	N	NA
Chong 2014	Y	Y	Y	Y	Y	Y	N	Y	?	Y	Y	N	N	N	N	Y	Y	Y	N	Y
Choi 2011	Y	Y	N	Y	Y	Y	NA	Y	Y	Y	Y	Y	NA	NA	Y	Y	Y	Y	N	Y, N
Deva 2011	Y	Y	N	Y	Y	Y	NA	Y	?	Y	Y	Y	NA	NA	Y	Y	Y	N	N	Y
Gao 2011	Y	Y	N	Y	Y	Y	NA	Y	Y	Y	Y	Y	?	?	N	Y	Y	Y	N	Y
Weiner 2011	Y	Y	N	Y	Y	Y	N	Y	?	Y	Y	Y	N	?	Y	Y	Y	Y	Y	N
Nitsch 2009	Y	Y	N	Y	Y	?	Y	Y	?	Y	?	N	N	Y	Y	?	N	Y	N	Y, N
BES	Y	Y	NA	NA	NA	NA	NA	Y	Y	NA	NA	NA	NA	NA	NA	NA	NA	NA	NA	Y
CIEMS	Y	Y	NA	NA	NA	NA	NA	Y	Y	NA	NA	NA	NA	NA	NA	NA	NA	NA	NA	Y
HDES	Y	Y	NA	NA	NA	NA	NA	Y	Y	NA	NA	NA	NA	NA	NA	NA	NA	NA	NA	Y
KNHANES	Y	Y	NA	NA	NA	NA	NA	Y	Y	NA	NA	NA	NA	NA	NA	NA	NA	NA	NA	Y
SCES	Y	Y	NA	NA	NA	NA	NA	Y	Y	NA	NA	NA	NA	NA	NA	NA	NA	NA	NA	Y
SiMES	Y	Y	NA	NA	NA	NA	NA	Y	Y	NA	NA	NA	NA	NA	NA	NA	NA	NA	NA	Y
SINDI	Y	Y	NA	NA	NA	NA	NA	Y	Y	NA	NA	NA	NA	NA	NA	NA	NA	NA	NA	Y
SNDREAMS	Y	Y	NA	NA	NA	NA	NA	Y	Y	NA	NA	NA	NA	NA	NA	NA	NA	NA	NA	Y
TMCS	Y	Y	NA	NA	NA	NA	NA	Y	Y	NA	NA	NA	NA	NA	NA	NA	NA	NA	NA	Y
UEMS	Y	Y	NA	NA	NA	NA	NA	Y	Y	NA	NA	NA	NA	NA	NA	NA	NA	NA	NA	Y

Abbreviations: BES = Beijing Eye Study; CIEMS = Central India Eye and Medical Study; HDES = Handan Eye Study; KNHANES = Korean National Health and Nutrition Examination Survey; N=No; NA=Not applicable; SiMES = Singapore Malay Eye Study; SINDI = Singapore Indian Eye Study; SCES = Singapore Chinese Eye Study; SNDREAMS = Sankara Nethralaya-Diabetic Retinopathy Epidemiology and Molecular Genetic Study; TMCS = Tsuruoka Metabolomics Cohort Study; UEMS = Ural Eye and Medical Study; Y=Yes; ?=Unclear; Color codes indicate the level of risk of bias across AXIS domains: green = low risk, yellow = unclear, red = high, gray = not applicable.

†<20% non-responder = low (No for bias); 20–30% non-responder = intermediate (Unclear for bias); >30% non-responder = high (Yes for bias).

### 3.3 Primary outcomes

#### 3.3.1 Prevalence of all-stage AMD

**Figure 2:**
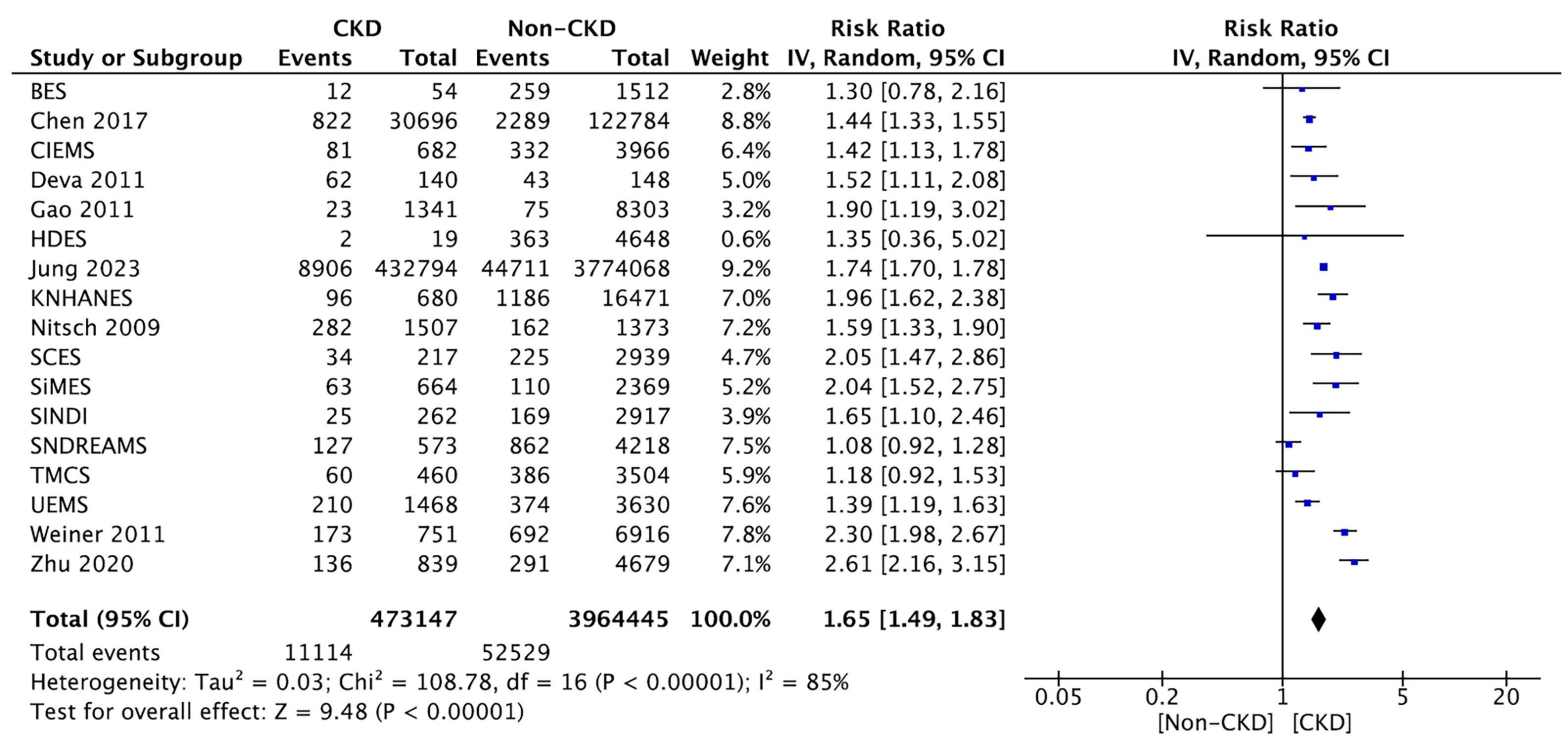
Forest plot of all-stage AMD prevalence comparing CKD and non-CKD populations. Risk ratios (RRs) with 95% confidence intervals showing a significantly higher prevalence of all-stage AMD in patients with CKD.

Seventeen studies compared between CKD and non-CKD patients on the prevalence of all AMD stages. The meta-analysis revealed a significantly higher prevalence of all AMD stages for CKD patients than for those without CKD (RR: 1.65, 95% CI: 1.49–1.83, *p* < 0.00001; Figure 2).

#### 3.3.2 Prevalence of early-stage AMD

**Figure 3:**
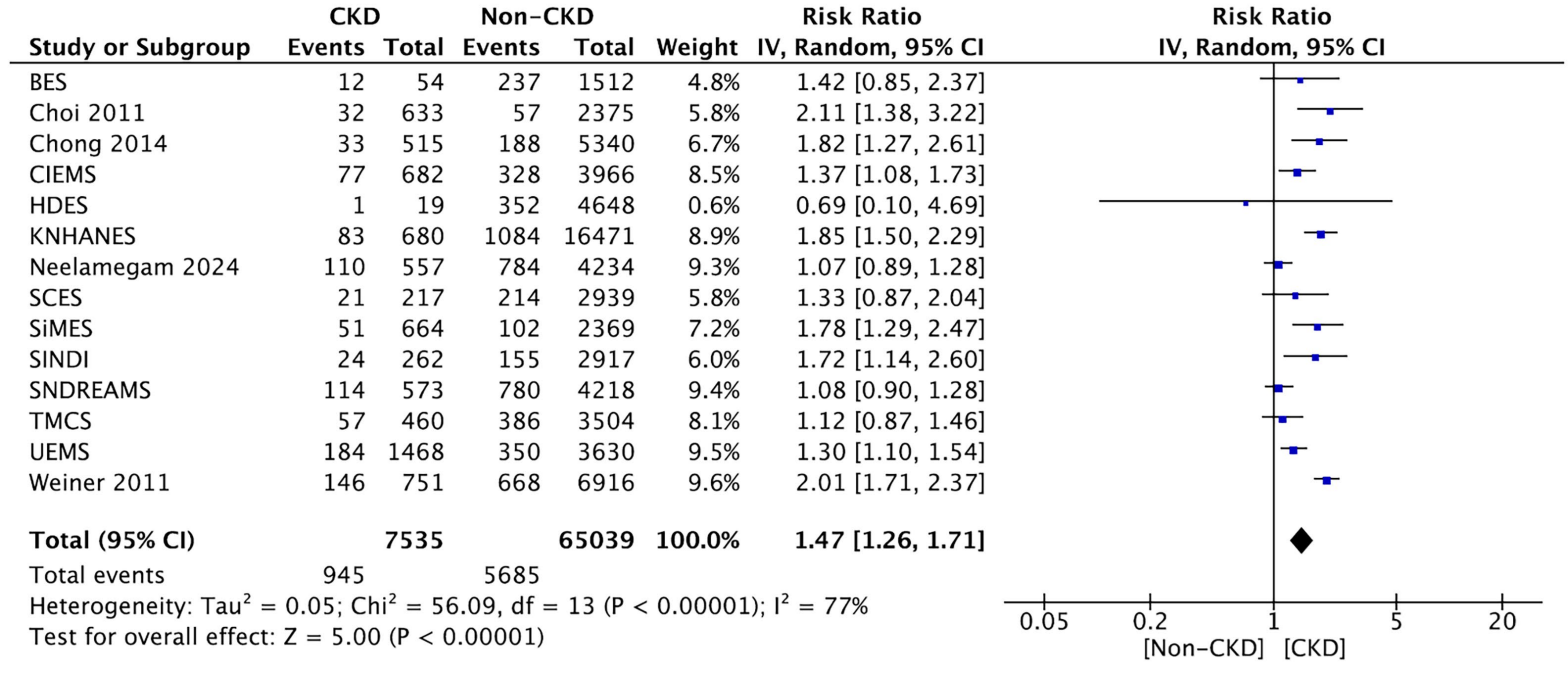
Forest plot of early-stage AMD prevalence comparing CKD and non-CKD populations. Meta-analysis results demonstrating increased early-stage AMD prevalence among CKD patients.

Thirteen studies compared between CKD and non-CKD patients on the prevalence of early-stage AMD. The meta-analysis revealed a significantly higher prevalence of early-stage AMD for CKD patients than for those without CKD (RR: 1.47, 95% CI: 1.26–1.71, *p* < 0.00001; Figure 3).

##### 3.3.2.1 Subgroup analyses

Stratified results showed higher pooled risk in advanced CKD (eGFR < 30 mL/min/1.73 m^2^) than in moderate CKD (eGFR 30–59), particularly for late-stage AMD. By geographical region, East-Asian cohorts exhibited a comparable direction of effect to non-Asian cohorts, with overlapping CIs. The results were consistent across study designs (population-based vs. clinic-based), and leave-one-out analyses did not materially alter pooled estimates.

#### 3.3.3 Prevalence of late-(advanced-)stage AMD

**Figure 4:**
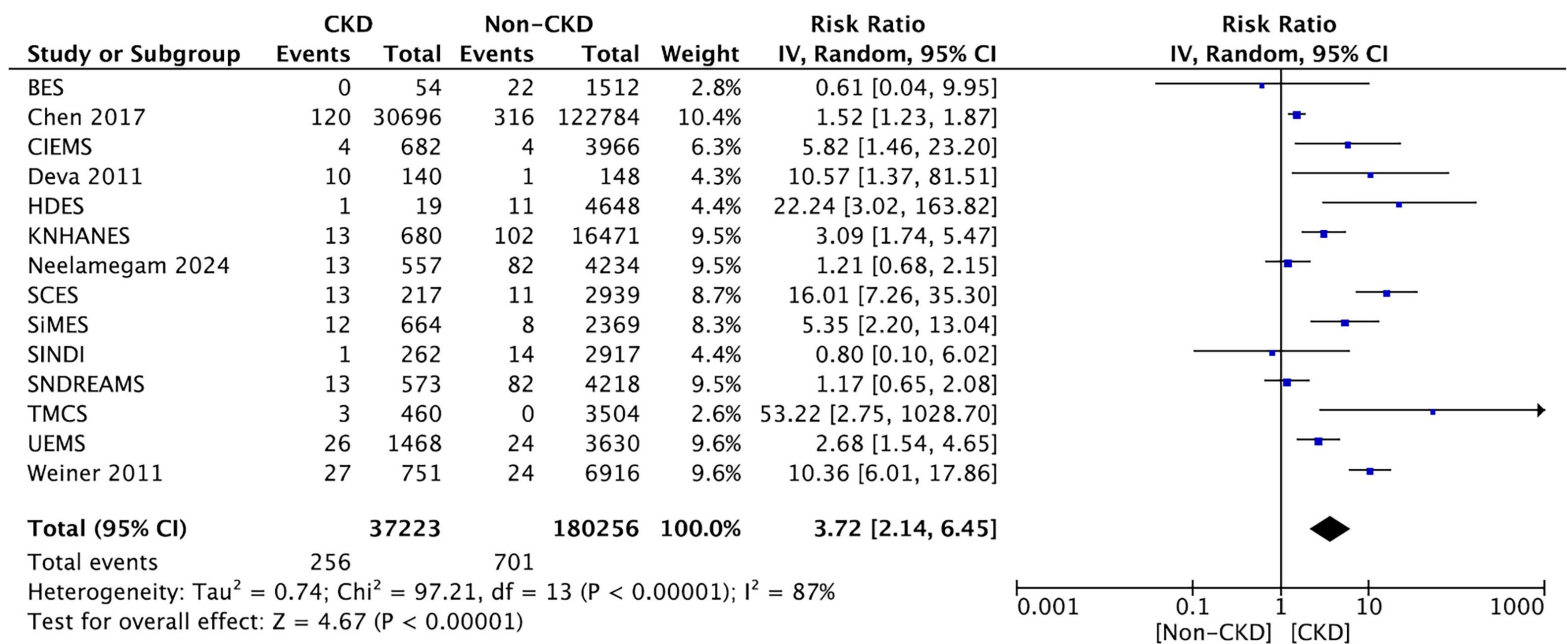
Forest plot of late-stage AMD prevalence comparing CKD and non-CKD populations. Summary of pooled RRs indicating a strong association between CKD and late-stage AMD.

Fourteen studies compared between CKD and non-CKD patients on the prevalence of late-stage AMD. The term “severe macular degeneration,” which was found in one study (19), is considered equivalent to “late AMD” in the current study. The meta-analysis revealed a significantly higher prevalence of late-stage AMD in CKD patients than those without CKD (RR: 3.72, 95% CI: 2.14–6.45, *p* < 0.00001; Figure 4).

### 3.4 Secondary outcomes

#### 3.4.1 Comparison between the moderate and advanced stages of CKD about the prevalence of AMD

**Figure 5:**
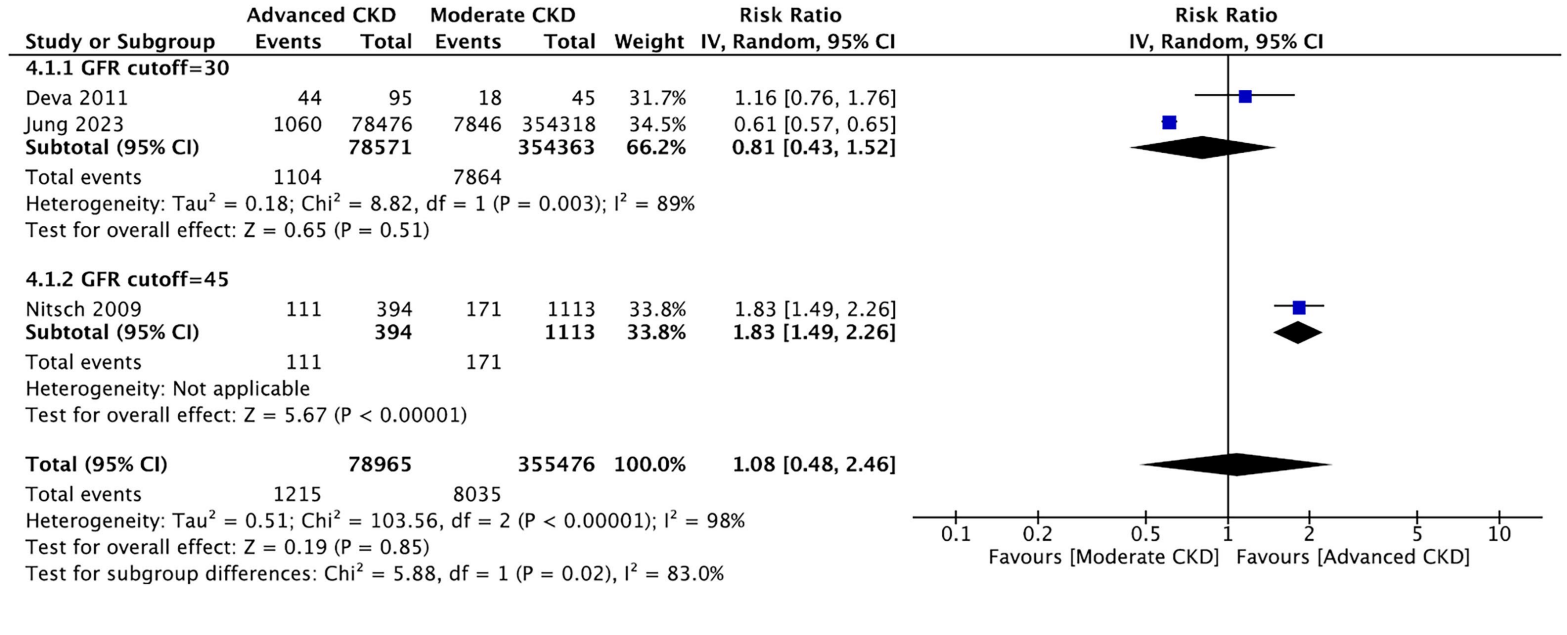
Comparison of AMD prevalence between moderate and advanced CKD stages. Forest plot showing no significant difference in AMD prevalence between moderate (eGFR 30–59) and advanced (eGFR <30) CKD groups.

Three studies compared between the moderate stage and advanced stages of CKD on the prevalence of AMD. We did not observe a significant difference in the prevalence of AMD between patients of moderate and advanced CKD stages (RR: 1.08, 95% CI: 0.48–2.46, *p* = 0.85; Figure 5). Sensitivity analyses excluding each study sequentially did not materially change the pooled estimates, indicating the robustness of the synthesized results. Additional analyses according to extracted variables (e.g., eGFR range, serum creatinine, and study region) demonstrated consistent directions of effect.

## 4 Discussion

The current systematic and meta-analytic review indicates a positive relationship between the diseases of CKD and AMD. Our results revealed that, regardless of the AMD stages, the positive status of CKD was associated with a higher prevalence of AMD.

Clinically, this association underscores the importance of cross-disciplinary disease surveillance between nephrology and ophthalmology. CKD patients frequently exhibit systemic inflammation, oxidative stress, and endothelial injury, all of which are also key contributors to retinal degeneration. Accordingly, regular ophthalmologic evaluations should be incorporated into the routine management of CKD, especially among elderly individuals or those with diabetes and hypertension. Early detection of subclinical AMD in this population may enable timely preventive interventions, such as lifestyle modification, antioxidant supplementation, and anti-inflammatory therapy, to delay disease progression.

From a mechanistic standpoint, CKD-related uremic toxins such as indoxyl sulfate and p-cresyl sulfate can penetrate the blood–retinal barrier, promote oxidative injury, and compromise retinal pigment epithelium integrity. Dysregulation of the complement pathway, particularly involving complement factor D, is implicated in both CKD-associated chronic inflammation and AMD pathogenesis. Furthermore, endothelial dysfunction and microvascular calcification, which are frequently observed in CKD, may reduce choroidal perfusion and induce retinal hypoxia, ultimately contributing to photoreceptor loss.

Collectively, these findings suggest that a multidisciplinary approach involving nephrologists and ophthalmologists is warranted. For patients with declining renal function, routine retinal imaging and visual function assessment may allow an earlier diagnosis of AMD. Conversely, ophthalmologists managing AMD should be aware of the underlying renal impairment, as systemic inflammation and metabolic imbalance could influence treatment response and disease progression.

In addition to the diagnostic and preventive perspectives, potential therapeutic implications can also be derived from the shared pathophysiology of CKD and AMD. Antioxidant and anti-inflammatory therapies have shown promise in attenuating oxidative injury in both renal and retinal tissues. Agents, such as N-acetylcysteine, vitamin E, and resveratrol analogs, may mitigate the accumulation of reactive oxygen species and improve endothelial function. Similarly, lifestyle modifications such as smoking cessation, dietary control, and optimization of blood pressure and glycemic status can synergistically protect both renal and ocular microvasculature.

Targeting the complement pathway is another emerging approach. Complement factor D inhibitors (e.g., lampalizumab and other anti-CFD biologics) have been demonstrated to reduce geographic atrophy progression in AMD, and comparable anti-complement strategies are under investigation in CKD to curb chronic inflammation. This overlap supports the notion that systemic complement regulation could simultaneously benefit both organs.

Moreover, endothelial-protective and renoprotective agents—such as SGLT2 inhibitors, renin–angiotensin system blockers, and nitric oxide–enhancing compounds—have been shown to improve vascular integrity in CKD populations, and such vascular benefits may extend to retinal microcirculation (30). Their potential to stabilize choroidal and retinal circulation may represent a promising therapeutic avenue for patients with dual CKD–AMD pathology.

Collectively, these findings emphasize that the management of CKD should not only be limited to renal endpoints alone but should also aim to preserve retinal health through systemic anti-oxidative and anti-inflammatory strategies.

Common risk factors have been proposed to explain the co-existence of CKD and AMD, one of which is the action of complements on the inflammatory process. The complement factor D (CFD) is an activator of the alternative complement pathway. As CFD elevation is considered to be linked to chronic inflammation in CKD (31), CFD being identified as a possible target in the treatment of AMD (32) should explain the correlation between CKD and AMD. Recent mechanistic and clinical evidence further supports complement dysregulation and systemic inflammation as shared drivers of CKD–AMD pathology. A bidirectional Mendelian randomization study demonstrated that elevated systemic inflammatory regulators are causally associated with a higher risk of AMD, reinforcing the role of chronic inflammation beyond local retinal processes (12). Dysregulated activation of the complement cascade—particularly complement factor D—links renal and retinal microvascular inflammation, providing a unifying explanation for their coexistence.

In addition, phase 3 clinical data have confirmed the translational relevance of this pathway: the OAKS and DERBY trials showed that complement C3 inhibition with pegcetacoplan significantly slowed the progression of geographic atrophy secondary to AMD over 24 months (33). These findings highlight that complement-targeted therapy can effectively attenuate retinal degeneration and suggest that modulating complement activity may also benefit patients with concurrent CKD by mitigating inflammation-driven microvascular injury.

Indoxyl sulfate (IS) and p-cresol sulfate (PCS) are two protein-bound uremic toxins in CKD. They are reported to generate oxidative stress (9), endothelial dysfunction (10) and vascular calcification (34). From a structural perspective, the blood–retinal barrier (BRB) is made up of retinal pigmented epithelium (RPE) cells and the retinal vessel endothelium. RPE cell functionality impairment, which is the hallmark of AMD, was indicated to arise from oxidative stress (11). Consistently, a bidirectional Mendelian randomization study linked systemic inflammatory regulators to AMD risk, reinforcing the plausibility that CKD-related systemic inflammation contributes to retinal degeneration (12). Where endothelial dysfunction is concerned, circulating endothelial cells, a potential biomarker for such a disorder (35), were suggested to possibly promote AMD (36). With regards to calcification, a pro-calcific environment hampers ischemia-driven angiogenesis (37). Thus, IS and PCS are expected to decrease angiogenesis, which, along with the subsequently decreased RPE cell survival, may lead to dry AMD (13). With the above considered, we speculate that the actions of IS and PCS are built on their ability to trespass the BRB, as a rat model reported that IS breaches the BRB and accumulates in the intraocular fluid (38), while another previous study showed that PCS damages the blood–brain barrier function, whose inner layer shares similarity with the BRB (39).

Another risk factor is the acceleration of atherosclerosis, which has been indicated in CKD due to multiple speculated mechanisms. These mechanisms include elevated serum homocysteine (40) and lipoprotein (41). With studies also supporting the connection between atherosclerosis and AMD (42, 43), it would be feasible to attribute the prevalence of AMD in CKD patients to the influence of atherosclerosis.

In the current review, no significant difference was observed in the prevalence of AMD between patients of moderate and advanced CKD stages (Figure 5), which is opposite to what we originally hypothesized. However, only three studies (16, 19, 20) were considered eligible for meta-analysis on this subtopic, and the current result appears to be lopsided by the large number of patients in one of the studies (16). Therefore, further analyses are required when more relevant studies that grade kidney functions become available.

To the best of our knowledge, there was only one previous meta-analysis that addressed the connection between CKD and AMD (14). Incorporating 12 observational studies (with 3 cohorts, 2 case controls, and 7 cross-sectionals), this study suggested a significant association between the 2 diseases.

There are several limitations to the current study. First, the majority of the included patients in our study are Asian, which restricted the generalizability of our article. Second, since we included only cross-sectional studies, the inherent weakness of our results is the causal inference. In addition, the review process was limited by the potential omission of gray literature and non-English-language publications, which may contribute to selection bias. Data extraction was performed by two reviewers, but no automation tools were used, which could introduce human error. Other causes that might result from the prevalence of AMD in the CKD population were not fully considered. Reviews that investigate the incidence of AMD in CKD are required to strengthen our findings. Finally, the influence of sex difference has not been investigated in the current study. The above limitations are expected to be addressed by future studies to provide a more comprehensive view of the current topic.

Furthermore, most of the included studies were conducted among Asian populations, which may limit the generalizability of our findings to other ethnic or geographic groups. Differences in genetic background, dietary habits, and environmental exposures could alter both the prevalence and the strength of the CKD–AMD association. For example, risk variants in the CFH and ARMS2 genes show different allele frequencies between Asian and Western populations, potentially affecting susceptibility to AMD and the inflammatory response related to CKD. Therefore, caution should be exercised when extrapolating our results to non-Asian populations. Future large-scale studies incorporating multi-ethnic and regionally diverse cohorts are warranted to validate these findings and enhance external validity.

Based on our findings from the current study, we concluded that CKD is a risk factor for AMD. Our findings highlight the need for closer ophthalmic surveillance in patients with chronic kidney disease, especially among those with declining eGFR or metabolic comorbidities. Early fundus screening may facilitate timely detection and intervention for AMD in this population. Clinicians should also be aware that systemic inflammation and vascular dysfunction observed in CKD could modify the response to AMD therapy. Future research should validate these associations through longitudinal and multi-ethnic cohorts, integrating biochemical markers (e.g., complement factors and uremic toxins) and imaging biomarkers to clarify causal pathways and optimize individualized management strategies.

## Data Availability

The original contributions presented in the study are included in the article/supplementary material, further inquiries can be directed to the corresponding author.
